# Fatal Suicidal Cut-Throat Wound in a Patient With Double Valve Replacement: Interplay of Cardiac Dysfunction and Self-Harm

**DOI:** 10.7759/cureus.84464

**Published:** 2025-05-20

**Authors:** Ajay K Bhagat, Kumar Shubhendu, Ankur Chaudhary, Anand Kumar

**Affiliations:** 1 Department of Forensic Medicine and Toxicology, Rajendra Institute of Medical Sciences, Ranchi, IND

**Keywords:** cardiac valve replacement, cut-throat injury, haemorrhagic shock, medicolegal autopsy, rheumatic heart disease, suicide

## Abstract

Suicidal incised neck injuries are infrequent and exhibit considerable forensic intricacies, particularly when compounded by pre-existing medical conditions. We delineate a case involving a 32-year-old male patient diagnosed with severe rheumatic heart disease and a history of double valve replacement, who succumbed to a self-inflicted incised neck wound during his hospitalization. The 7.5-cm incised wound presented two skin tags, indicative of hesitation cuts, and was devoid of any defensive injuries. The internal examination indicated an enlarged heart, artificial heart valves in situ, a pale appearance of the organs, and haemorrhagic infiltration around the wound; anatomical parts like the trachea and oesophagus were intact. The mechanism of mortality was ascribed to be haemorrhagic shock. The patient's impaired cardiac function likely contributed to the expedited fatal outcome by restricting physiological compensatory mechanisms. This case exemplifies the complex interaction between chronic illness and suicidal tendencies, underscoring the need for comprehensive forensic analysis and proactive mental health assessments in individuals with medical vulnerabilities.

## Introduction

Self-inflicted incised wounds to the neck, commonly termed "cut-throat injuries," represent an uncommon yet striking manifestation of self-directed injury, carrying substantial clinical and forensic significance. These kinds of injuries account for a minimal share of international suicide figures, with alternative means like hanging, poisoning, self-immolation, and firearms being notably more common [1,2]. Nonetheless, their occurrence demands an exhaustive forensic analysis owing to the risk of misclassification as homicides, particularly when the characteristics of the wounds or the evidence at the scene lack clarity. The notable risk related to neck injuries stems from the collection of key anatomical parts in the cervical region, which involves the carotid arteries, jugular veins, trachea, oesophagus, and spinal cord [3,4]. Therefore, a rigorous forensic methodology is essential for the evaluation of wound morphology, directional attributes, depth, and accompanying features like hesitation marks or signs of vital reactions.

The forensic differentiation between suicidal and homicidal neck injuries is often complex. Suicidal wounds typically exhibit features such as a single deep incised wound, associated hesitation cuts or "tentative wounds," a predictable wound trajectory, and an absence of defensive injuries. In contrast, homicidal neck wounds may be multiple, more forceful, and accompanied by signs of struggle or restraint, as well as injuries to other parts of the body. Additionally, in suicidal cases, the weapon is frequently found near the body, and the act is often carried out in privacy or isolation [5,6].

Psychological and psychiatric determinants are integral to understanding suicidal behaviour. Empirical investigations indicate that individuals experiencing depression, psychosis, substance dependence, or chronic medical ailments exhibit a markedly elevated risk of suicide [7,8]. Within populations afflicted by medical conditions, those diagnosed with chronic cardiac diseases, particularly those characterized by heart failure or structural valvular impairments, exhibit a significant incidence of depression and suicidal ideation. The burden of physical ailments such as dyspnoea, fatigue, impaired mobility, and the psychological turmoil linked to a terminal diagnosis engender feelings of despair, which may lead to suicidal crises in certain instances [9,10]. In these scenarios, suicidality may manifest either impulsively or as an ostensibly rational means of escaping suffering.

People with earlier heart surgical procedures, particularly those involving prosthetic valves, commonly deal with ongoing complications and symptoms that can harm their general quality of life. Complications associated with the valves, requirements for anticoagulation therapy, arrhythmias, and recurrent hospital admissions, often exacerbated by financial limitations, may further impair emotional well-being. The correlation between persistent physical ailments and suicidal tendencies highlights the necessity for comprehensive care models that concurrently address both physiological and psychological health in the context of long-term disease management [11,12].

In hospital or institutional settings, suicides are particularly concerning due to questions surrounding supervision, access to means, and preventive measures. Cut-throat suicides occurring within a hospital environment are extremely rare and can raise suspicion of foul play unless carefully evaluated. These situations demand detailed investigation not only of the autopsy findings but also of the behavioural observations, psychiatric history, accessibility of instruments, and environmental controls in place at the time of the incident.

This case study details the uncommon and clinically intricate circumstance of a 32-year-old male patient, who has a history of severe rheumatic heart disease and a prior double valve replacement, who succumbed to a self-inflicted incised wound to the neck while hospitalized. The case illustrates the intricate nature of suicide, notably among those enduring persistent health challenges, and underlines the need for a detailed forensic, clinical, and psychological appraisal in identifying the cause of death. This case study was presented at the 46th Annual National Conference of the Indian Academy of Forensic Medicine (Forensic Medicon 2025) on January 31, 2025.

## Case presentation

Case background

The deceased was a 32-year-old male patient admitted to the general wards, displaying indications of a lasting cough, worsening difficulty in breathing, and an inability to lie flat (orthopnoea). His medical background notably included rheumatic heart disease, characterized by serious regurgitation issues with the aortic and mitral valves, moderate tricuspid regurgitation, and compromised left ventricular function.

**Figure 1:**
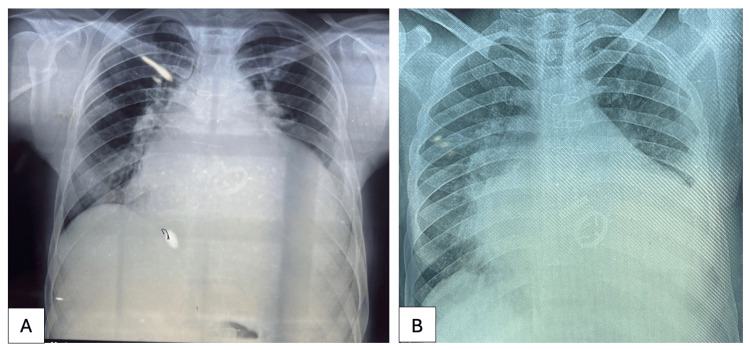
Chest radiographs: posteroanterior view A. Two years after the cardiac surgery (depicting Gross cardiomegaly, sternal sutures and prosthetic cardiac valves in situ) B. Four years and five months after the cardiac surgery (depicting Gross cardiomegaly, sternal sutures and prosthetic cardiac valves in situ, along with ground-glass opacities in bilateral lung fields)

**Figure 2:**
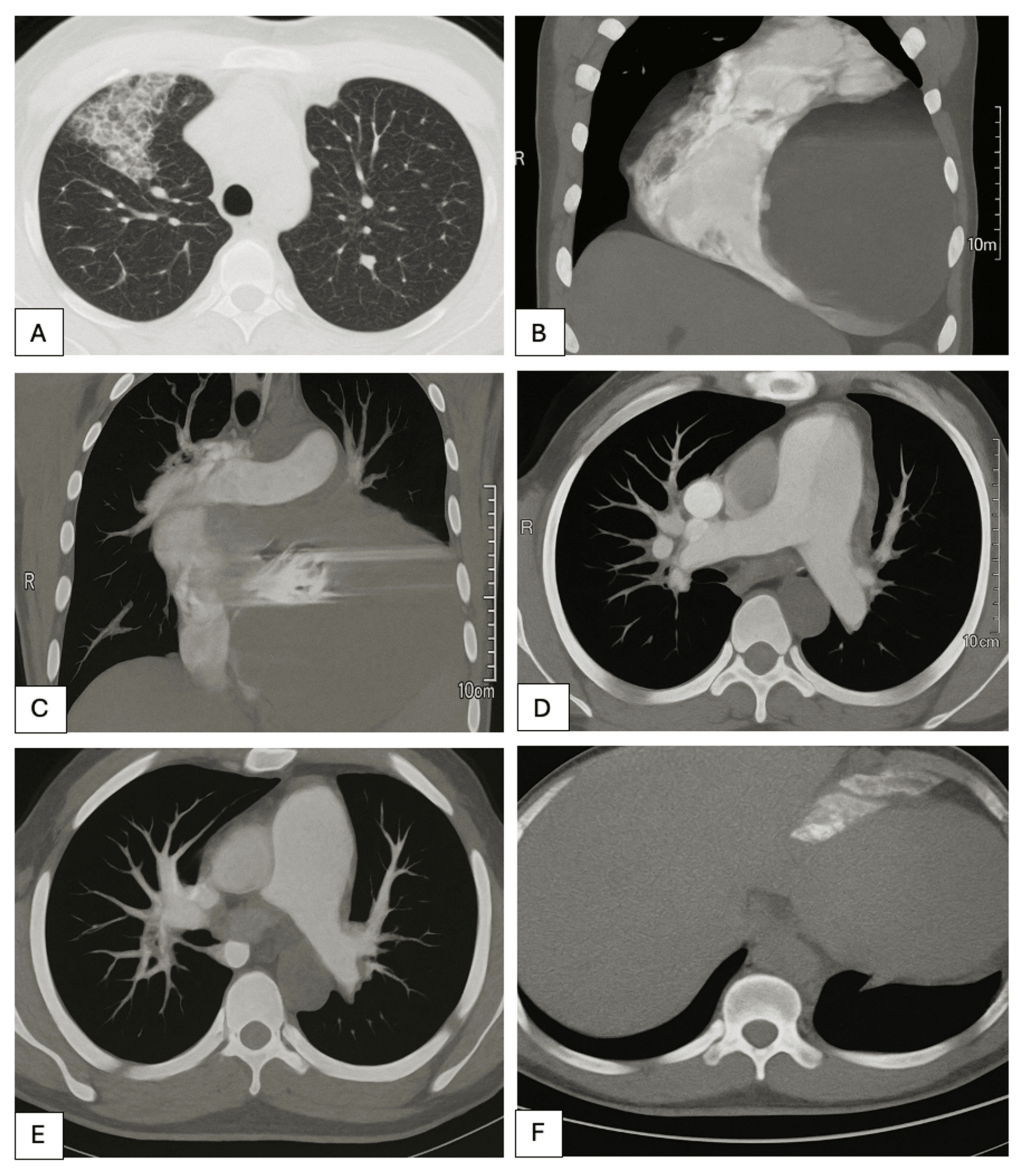
Computed tomography pulmonary angiogram performed two months prior to the death of the deceased patient. A. There was evidence of ground-glass opacity in apico-anterior segment of upper lobe of right lung, likely alveolar haemorrhages. B. Gross cardiomegaly was noted with maximally dilated left ventricle. Left atrium was also dilated. C. Prosthetic cardiac valves were seen in situ. D. There was dilatation of main pulmonary artery, as well as left and right pulmonary arteries suggesting pulmonary hypertension. No filling defect was seen. E. Segmental and sub-segmental pulmonary arteries also did not show any filling defect. There was no evidence of thromboembolism. F. Inferior vena cava was prominent.

Approximately 4.5 years prior to his demise, the patient had undergone major cardiac surgery, which entailed a double valve replacement utilizing TTK Chitra prosthetic valves (aortic 29 mm and mitral 33 mm), left atrial appendage ligation, and De Vega’s repair of the tricuspid valve. The TTK Chitra prosthetic heart valve was designed and developed by Sree Chitra Tirunal Institute for Medical Sciences and Technology (SCTIMST), Thiruvananthapuram, Kerala, India and manufactured by Heart Valve Division, TTK Healthcare Limited, Thumba, Thiruvananthapuram, Kerala, India. Notwithstanding the surgical intervention, he continued to exhibit symptoms (Figures 1, 2) and was reportedly suffering from psychological distress characterized by sensations of impending doom and despair. In the early morning hours, he was found lifeless in the hospital ward, with a kitchen knife near his body and a deep incised wound on his neck.

Autopsy findings

External Examination

**Figure 3:**
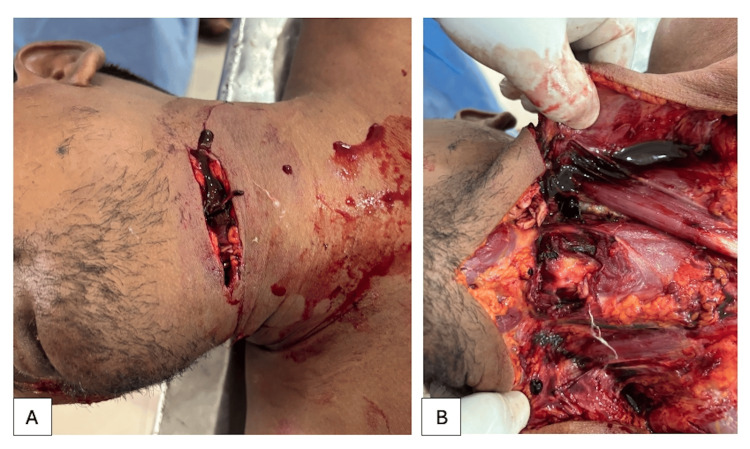
Autopsy findings in the neck A. Incised wound over the upper left fronto-lateral aspect of the neck. B. Deep incised wound involving the skin, subcutaneous tissues, and muscles. Major blood vessels were severed, but the trachea and oesophagus were spared.

An incised wound measuring 7.5 cm was noted over the upper left fronto-lateral aspect of the neck. The presence of two skin tags suggested at least three separate attempts to inflict the injury, a characteristic feature of tentative or hesitation wounds often seen in suicides (Figure 3A). Additionally, two old surgical scars were present: one measuring 18 cm over the anterior midline of the chest (consistent with sternotomy), and another smaller scar (3 cm) over the upper abdomen. Notably, no defensive wounds were observed on the upper limbs or elsewhere on the body.

Internal Examination

**Figure 4:**
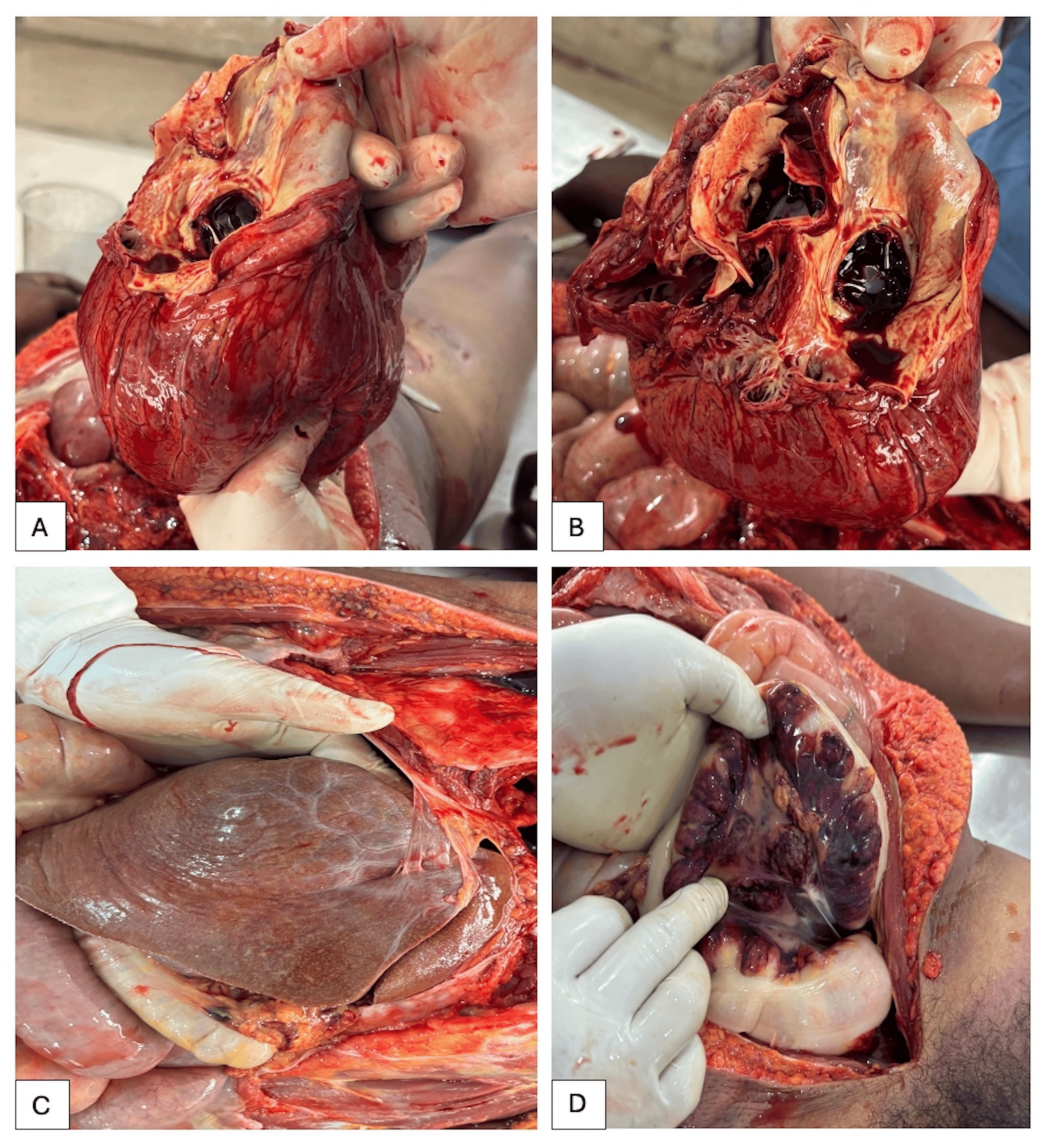
Thoraco-abdominal autopsy findings A. Gross cardiomegaly. B. Prosthetic mitral and aortic valves. C. Hepato-diaphragmatic adhesions. D. Mesenteric ischaemic changes along with necrosis of descending colon, and sigmoid colon.

Dissection of the neck uncovered a deep incised wound affecting the integumentary layer, subcutaneous structures, and musculature. Significant vascular structures were transected, yet both the trachea and oesophagus remained intact (Figure 3B). The observation of haemorrhage and vital responses substantiated that the inflicted trauma occurred prior to death. The sternum bore evidence of a prior surgical incision fixed with steel wires. Both lungs exhibited adherence to the thoracic wall, while the pericardium demonstrated adherence to the adjacent mediastinal structures. The heart was massively enlarged, weighing approximately 1300 grams, and displayed marked hypertrophy (Figure 4A). Prosthetic valves were observed in both the mitral and aortic positions (Figure 4B). Additional findings included moderate ascites, hepatodiaphragmatic adhesions (Figure 4C), splenomegaly, and necrosis involving the mesentery, descending colon, and sigmoid colon (Figure 4D). All internal organs appeared pale, indicative of significant blood loss.

Cause of Death

The immediate cause of death was determined to be haemorrhagic shock due to a self-inflicted cut-throat injury using a sharp cutting cum pointed weapon. The underlying chronic cardiac disease, with severely compromised cardiac function, likely played a contributory role by hastening the fatal outcome due to reduced physiological reserve. The mechanism of death was exsanguination, leading to cardiac arrest.

Forensic Interpretation

The wound pattern, its location, depth, and the presence of skin tags strongly supported a self-inflicted injury. The absence of defensive wounds or signs of struggle, along with the presence of the weapon nearby and lack of evidence indicating third-party involvement, confirmed the manner of death as suicide. Psychological elements, as derived from history given by relatives portrayed a sense of impending doom and emotional burden, likely contributing to the suicidal act. The chronic and progressive nature of his heart disease, coupled with the limitations in daily functioning and persistent symptoms, appeared to have significantly impacted his mental well-being. The physical debilitation may have served as a catalyst for suicidal ideation, particularly in a hospital setting where access to means and absence of constant supervision could facilitate such actions.

## Discussion

Suicidal incised wounds are frequently marked by significant anatomical trauma and the possibility of misidentification due to their similarity to injuries resulting from homicide. From a forensic perspective, a meticulous assessment of the characteristics of the wounds, the psychiatric background, and the surrounding circumstances is crucial in establishing the manner of death [5,6,13]. In this case, the deceased individual, a 32-year-old male patient, suffered from long-term rheumatic heart complications, highlighted by significant aortic and mitral valve leakage, complicated by prior heart operations. Individuals with long-term cardiovascular ailments typically endure compromised hemodynamic stability, diminished functional capabilities, and ongoing psychological stressors stemming from frequent hospital admissions, the burden of pharmacotherapy, and apprehension regarding potential deterioration or mortality [11,12]. These elements can profoundly influence mental well-being, rendering individuals susceptible to depressive disorders and, in extreme instances, suicidal thoughts.

Research has consistently indicated a robust correlation between chronic physical ailments and an elevated risk of suicide. Notably, cardiovascular disease has been recognized as an autonomous risk factor for suicidal behaviour, particularly among younger demographics who may possess limited coping strategies or insufficient psychosocial support [14,15]. Individuals suffering from heart failure demonstrate a suicide incidence that is nearly double that of the general populace, a phenomenon attributed to perceptions of disability, persistent pain, feelings of hopelessness, and social isolation [16]. The concept of "rational suicide," in which individuals confronting irreversible deterioration and suffering make a conscious decision to terminate their lives, may also be relevant in this context, especially when taking into account the patient’s expressed sense of doom and declining health status [17].

From a forensic perspective, the injury characteristics support a suicidal origin. The presence of a single incised wound with two skin tags suggests multiple tentative efforts before the fatal incision, a classical feature of self-inflicted injuries. The absence of defence wounds and signs of struggle further supports the conclusion that the act was self-perpetrated. Moreover, the location (left fronto-lateral neck), directionality, and sparing of vital structures such as the trachea and oesophagus, are commonly observed in suicidal cut-throat wounds [5,6]. The presence of haemorrhage and vital reaction confirms the antemortem nature of the wound, strengthening the interpretation. Furthermore, the subject's cardiac dysfunction likely contributed to the mechanisms and speed of demise. The heart exhibited pronounced hypertrophy, suggesting considerable chronic volume overload and myocardial stress. The diminished cardiovascular reserve would have significantly restricted the physiological ability to endure blood loss. In such scenarios, even a minor haemorrhage can swiftly precipitate hypovolemic shock and mortality. 

The psychological component is equally critical. As per history given by the relatives, the patient's reported insomnia, breathlessness, and feelings of impending doom are consistent with depressive or anxiety-spectrum symptomatology, which are known precursors to suicidal behaviour [9,10]. In hospital settings, suicidal behaviour is often underestimated, particularly in non-psychiatric wards. Easy access to sharp instruments (like kitchen knives), lack of surveillance during early morning hours, and failure to recognize early psychological warning signs may contribute to such tragic outcomes [18]. Hospital-based suicides, especially those involving violent methods, raise important questions about patient safety protocols. Literature underscores the need for routine psychological screening in patients with chronic and terminal illnesses, especially those demonstrating behavioural changes, withdrawal, or expressions of hopelessness. Suicide prevention in medical wards should incorporate environmental risk assessments, staff training, and multidisciplinary collaboration to ensure early detection and intervention [19,20].

This particular case underscores the critical forensic significance of reconstructing the scene and meticulous documentation. In cases involving solitary cut-throat injuries, the synthesis of clinical history, wound characteristics, autopsy results, and psychological assessments is essential for precise interpretation. This comprehensive methodology aids in ruling out foul play, validating instances of suicide, and enhancing both medicolegal determinations and the public health comprehension of suicide among medical patients.

## Conclusions

This case exemplifies the intricate relationship between persistent physical illness and suicidal behaviour, emphasizing the necessity for a sophisticated approach to both clinical treatment and forensic analysis. The deceased, a young male patient afflicted with severe rheumatic heart disease and a history of valve replacement, epitomizes a vulnerable demographic frequently neglected in terms of psychological assistance. His choice to terminate his life using an uncommon and violent method, namely a self-inflicted cut-throat injury, highlights the profound psychological anguish that can accompany chronic cardiac ailments. From a clinical perspective, this case underscores the imperative for comprehensive patient management in contexts of chronic illness. Psychological assessment, suicide risk stratification, and the implementation of suitable safety protocols are vital components of inpatient care, particularly for individuals exhibiting emotional distress or observable behavioural alterations. The absence of these measures can result in tragic yet avoidable consequences, even in closely monitored medical settings.

This case ultimately emphasizes the hidden challenges associated with chronic health issues and the urgent need to focus on both the physical and mental health elements of patient treatment. Effective suicide prevention must progress from reactive interventions to a proactive, integrated strategy that emphasizes early identification, mental health resources, and ongoing monitoring of at-risk individuals.
